# Compliance-Free ZrO_2_/ZrO_2 − *x*_/ZrO_2_ Resistive Memory with Controllable Interfacial Multistate Switching Behaviour

**DOI:** 10.1186/s11671-017-2155-0

**Published:** 2017-06-02

**Authors:** Ruomeng Huang, Xingzhao Yan, Sheng Ye, Reza Kashtiban, Richard Beanland, Katrina A. Morgan, Martin D. B. Charlton, C. H. (Kees) de Groot

**Affiliations:** 1 Nanoelectronics and Nanotechnology Group, Department of Electronics and Computer Science, University of Southampton, Southampton, SO17 1BJ UK; 2 Department of Physics, University of Warwick, Coventry, CV4 7AL UK

**Keywords:** Resistive random access memory, Interfacial switching, Multistate, Compliance-free

## Abstract

A controllable transformation from interfacial to filamentary switching mode is presented on a ZrO_2_/ZrO_2 − *x*_/ZrO_2_ tri-layer resistive memory. The two switching modes are investigated with possible switching and transformation mechanisms proposed. Resistivity modulation of the ZrO_2 − *x*_ layer is proposed to be responsible for the switching in the interfacial switching mode through injecting/retracting of oxygen ions. The switching is compliance-free due to the intrinsic series resistor by the filaments formed in the ZrO_2_ layers. By tuning the *RESET* voltages, controllable and stable multistate memory can be achieved which clearly points towards the capability of developing the next-generation multistate high-performance memory.

**Electronic supplementary material:**

The online version of this article (doi:10.1186/s11671-017-2155-0) contains supplementary material, which is available to authorized users.

## Background

The development of denser, faster and less energy-consuming non-volatile memory is of great importance to innovations in modern information technology [1, 2]. While many contenders have emerged to be the next generation of memory device, resistive random access memory (RRAM) based on metal oxides is one of the most promising candidates for its advantages of high speed, high scalability, low power consumption and good compatibility with the CMOS process [3]. Although the detailed switching mechanism of the resistive memory remains uncertain, it is widely accepted that the migration of oxygen vacancies under an applied electrical field plays a key role in the switching behaviour [4]. Depending on the switching mechanism, the resistive switching can be classified into filamentary and interfacial (homogeneous) modes. The filamentary mode is achieved by the formation and rupture of oxygen vacancy filament(s) between two electrodes. On the other hand, the resistance switching in the interfacial mode is controlled by the distribution of oxygen vacancies along an interface. The current is localized in the conducting filaments(s) in the the filamentary mode, while is distributed homogenerously across the device area in the interfacial mode [5, 6]. The resistive switching mode characterized in a memory device is strongly dependent on its structure. In general, the exhibition of the interfacial mode in the metal oxide-based system relies on the existence of an oxygen gradient profile along the vertical axis [7, 8]. Recently, the coexistence of both modes in one material system has also been reported [9–11]. By modulating the measurement parameters, transformation between these two modes can also be achieved [12]. However, the switching in the interfacial mode is usually attributed to the change of Schottky barrier height induced by the accumulation and depletion processes of carriers through defective states at the electrode/function layer interface [7, 9–18] rather than the effect of oxygen vacancies migration at the oxide/oxide interfaces.

Over the past few years, tremendous progress has been made to increase the storage density of RRAM. Apart from the efforts on scaling down the physical dimensions of memory cell, utilizing the intermediate-resistance states (IRS) between the high-resistance state (HRS) and low-resistance state (LRS) to realize multistate storage within one memory cell has become a popular alternative solution [19, 20]. This multistate storage behaviour is important for high-density storage and oxide-based electronic synaptic devices [21–23]. Multistate storage in filamentary switching mode is realized by controlling the width and/or number of conducting filaments with different *RESET* voltages or *SET* current compliances. A variety of metal oxides, including TiO_x_, ZnO, SiO_x_ and HfO_2_, have demonstrated multistate behaviour in RRAM devices [24–27]. Multistate storage in interfacial switching mode was also reported where the adjustment of the oxygen-defective region widths was proposed to be responsible for this behaviour [12, 28]. However, both switching modes require current compliance in the *SET* process to protect the device from breakdown and, in the case of filamentary switching, to achieve multiply lower resistance states. These requirements could lead to complexity in RRAM circuit design. Compliance-free resistive memory with controllable multistate switching behaviour could therefore be advantageous as it minimizes the current overshoot during switching and has the potential to greatly lower the fabrication cost [29, 30].

Recently, the usage of ZrO_2_ as the active switching layer has attracted attention because of its high thermodynamic stability, simple composition and semiconductor process compatibility [31–33]. In addition, inserting an alien layer within the ZrO_2_ film to produce an oxide heterostructure has been proved to be an effective method to improve the switching characteristics in ZrO_2_-based devices [34, 35]. In this work, we demonstrate a ZrO_2_/ZrO_2 − *x*_/ZrO_2_-based resistive memory in which an unstoichiometric ZrO_2 − *x*_ layer is formed within the ZrO_2_ layer by inserting a Zr layer. Both interfacial and filamentary modes are observed, and a controllable transformation from interfacial to filamentary can be realized. The switching characteristics and the performance for both modes are investigated. While the oxide/electrode interface has an effect on the switching behaviours in the filamentary mode, the switching in the interfacial mode strongly relied on the oxide/oxide interfaces in the tri-layer structures. Most importantly, the resistive switching under the interfacial mode enjoys a build-in compliance-free property as well as multistate storage behaviour under different *RESET* voltages.

## Methods

ZrO_2_ thin films were prepared by medium-frequency plasma-assisted magnetron sputtering (Leybold Optics HELIOS Pro XL) at room temperature. In this process, the substrate was rotating at a speed of 180 rpm to ensure a uniform deposition. During each rotation, a thin layer of Zr was firstly deposited from a Zr metal targets (99.99% purity) using a power of 2000 W in an Ar atmosphere. This thin film was transformed into an oxide layer by passing the substrate underneath the O_2_ plasma of the RF source. The O_2_ flow rate can be adjusted to produce ZrO_x_ films with different compositions. The compositional properties of the deposited films were investigated using energy-dispersive X-ray (EDX) where a Zeiss EVO LS 25 microscope equipped with an Oxford INCA x-act X-ray detector was used. Films with a large thickness of 1 μ m were deposited directly onto Si wafers to minimize the influence from the substrate. X-ray diffraction (XRD) patterns were collected in grazing incidence (*θ* = 1°) using a Rigaku Smartlab diffractometer with a 9-kW Cu–K_α_ source. X-ray photoelectron spectroscopy (XPS) data were obtained using a ThermoScientific Theta Probe System with Al–K_α_ radiation (photon energy = 1486.6 eV). XPS depth profile was performed by using an Ar ion gun at a beam voltage of 3 kV on a 2 × 2 mm raster area. Transmission electron microscopy (TEM) specimens were prepared using conventional mechanical polishing followed by ion milling to electron transparency using Ar+ at 6 keV. A final low-energy milling step was performed at 500 eV. In order to minimize surface damage, the structure and morphology of the samples were analysed using a JEOL 2100 TEM equipped with LaB_6_ and JEOL ARM200F TEM/scanning TEM (STEM) with a Schottky gun both operating at 200 kV. Annular dark-field (ADF) STEM measurement was performed in ARM200F, with probe and image aberration CEOS correctors. ADF-STEM images were obtained using a JEOL annular field detector with a probe current of approximately 23 pA, a convergence semi-angle of ∼25 mrad, and an inner angle of 45–50 mrad. An Oxford Instruments X-Max^N^ 100TLE windowless silicon drift detector (SSD) was used to perform STEM-EDX analysis.

The resistive switching behaviour of the tri-layer ZrO_2_/ZrO_2 − *x*_/ZrO_2_ film was investigated as memory. A 200-nm-thick TiN film was reactively sputtered (Ti target in a N_2_ atmosphere) onto the SiO_2_ layer to form the bottom electrode. This was followed by reactive sputtering of a second SiO_2_ layer (Si target in an O_2_ atmosphere). This layer of SiO_2_ was patterned to form active device areas by photolithography and reactive ion etch. Subsequently, ZrO_2_/ZrO_2 − *x*_/ZrO_2_ (20 nm/5 nm/20 nm) tri-layers were deposited to form the switching layer. The tri-layer structure was obtained by stopping the oxygen plasma during the ZrO_2_ growth. ZrO_2_ layer is achieved under an O_2_ flow rate of 20 sccm while the ZrO_2 − *x*_ layer is obtained by switching off the O_2_ flow for 20 s. An identical ZrO_2_/ZrO_2 − *x*_/ZrO_2_ tri-layer was also deposited on Si substrate for XRD and XPS characterization. Finally, a 200-nm TiN layer was sputtered and patterned on the tri-layer to form the top electrode. All electrical measurements were performed with a Keithley 4200 semiconductor characterization system. During the measurements, the programming voltage bias was applied to the top electrode, while keeping the bottom electrode grounded. The probe/point contacts to the top and bottom electrodes of the devices were realized through a pair of Wentworth probe needles, using a Wentworth laboratories AVT 702 semi-automatic prober. The voltage DC sweep measurements were conducted at a constant rate of 0.5 V/s.

## Results and Discussion

**Fig. 1:**
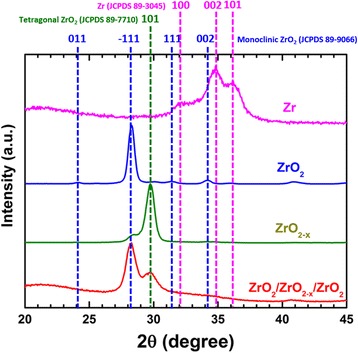
XRD patterns of the as-deposited ZrO_2_/ZrO_2 − *x*_/ZrO_2_ tri-layer structure (*red*), oxygen-deficient ZrO_2-*x*_ layer (*green*), stoichiometric ZrO_2_ layer (*blue*) and pure metallic Zr layer (*pink*)

The properties of the tri-layer structure were firstly investigated by XRD. Figure 1 depicts the XRD pattern of the as-deposited tri-layers (red) which features two peaks positioned at 28.2° and 29.8°. These two peaks can be assigned to the −111 peak from the monoclinic ZrO_2_ phase and the 101 peak from the tetragonal ZrO_2_ phase, respectively, indicating the existence of two phases. EDX and XRD characterizations carried out on single ZrO_*x*_ layers with different compositions (shown in Additional file 1: Figure S1 and S2) reveal that the stoichiometric ZrO_2_ displays the monoclinic phase (blue) while the tetragonal phase (green) was detected from the oxygen-deficient ZrO_2 − *x*_ layer. No XRD peaks of metallic Zr (pink) were observed in the tri-layer XRD pattern. This suggests the coexistence of both stoichiometric ZrO_2_ and oxygen-deficient ZrO_2 − *x*_ layer in the tri-layer structure and the embedded Zr layer has been oxidized.

**Fig. 2:**
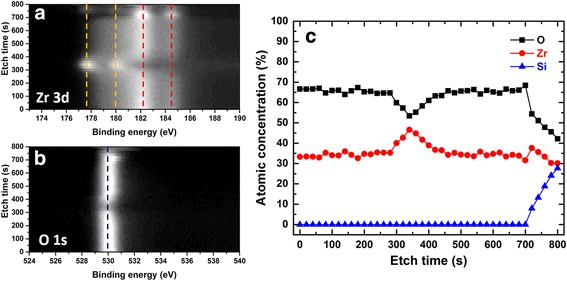
XPS spectra of **a** Zr 3d and **b** O 1s for the ZrO_2_/ZrO_2 − *x*_/ZrO_2_ tri-layer structure over an etch time of 700 s. **c** XPS depth profile of the ZrO_2_/ZrO_2 − *x*_/ZrO_2_ tri-layer structure

Figure 2a, b present the XPS results of Zr 3d and O 1s peak profiles over an etch time of 800 s. Two peaks positioned at ca. 182.3 and 184.5 eV can be ascribed to the fully oxidized Zr^4+^ state [36, 37], which dominate the Zr spectra up to an etch time of ca. 300 s. A clear enhanced intensity of two peaks positioned at ca. 178.3 and 180.5 eV can subsequently be observed between the etch time of 300 to 400 s; this is also accompanied by the reduction of O^2−^ peak intensity at ca. 530.0 eV. It has been suggested that these two Zr 3d peaks are associated with the metallic or non-oxidized Zr^0^ state [36]. This indicates the oxygen-deficient ZrO_2 − *x*_ layer lies in the middle of this tri-layer structure. After 400 s of etch time, the Zr^4+^ peaks resume their dominance and the intensity of the O^2−^ peak is back to normal. The atomic concentration along the depth profile in Fig. 2c further confirms the oxidization of the embedded Zr layer into non-stoichiometric ZrO_2 − *x*_. It is also worth mentioning that composition gradients between ZrO_2_ and ZrO_2 − *x*_ interfaces were also observed which is suggested to facilitate the formation of interfacial switching behaviour [8]. Considering both the XRD and XPS results, it is reasonable to believe that the monoclinic phase detected in the XRD is originated from the two stoichiometric ZrO_2_ layers. The sandwiched oxygen-deficient ZrO_2 − *x*_ layer, on the other hand, contributes to the tetragonal phase although some traces of amorphous regions cannot be ruled out.

**Fig. 3:**
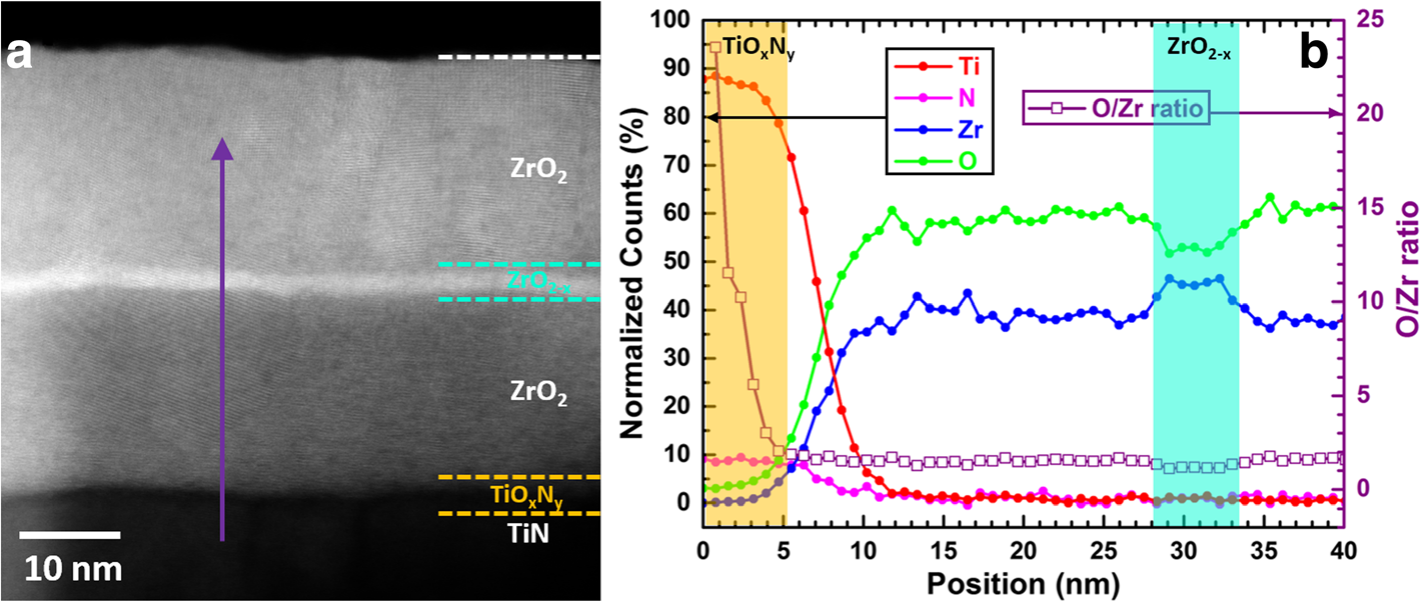
**a** ADF-STEM image of the cross section of the sample and **b** STEM-EDX elemental line profiles of the TiN/ZrO_2_/ZrO_2 − *x*_/ZrO_2_ structure

TEM measurement further confirms the tri-layer structure with the oxygen-deficient ZrO_2 − *x*_ layer clearly observed as shown in Fig. 3a. Furthermore, another interfacial layer between the ZrO_2_ layer and bottom TiN electrode is also visible. The corresponding EDX profile is demonstrated in Fig. 3b in which inter-diffusion of Ti, O, N and Zr atoms are evident in the first 10 nm. Moreover, the much higher O:Zr ratio (open square) in the first 5 nm confirms the existence of an TiO_*x*_N_*y*_ interfacial layer between the ZrO_2_ and the TiN bottom electrode. Indeed, as ZrO_2_ is sputtered immediately after TiN, the O_2_ plasma will react with the TiN surface to form a TiO_*x*_N_*y*_ layer when the ZrO_2_ layer is still very thin. Similar formation of interfacial TiO_*x*_N_*y*_ layer was also reported [38, 39].

**Fig. 4:**
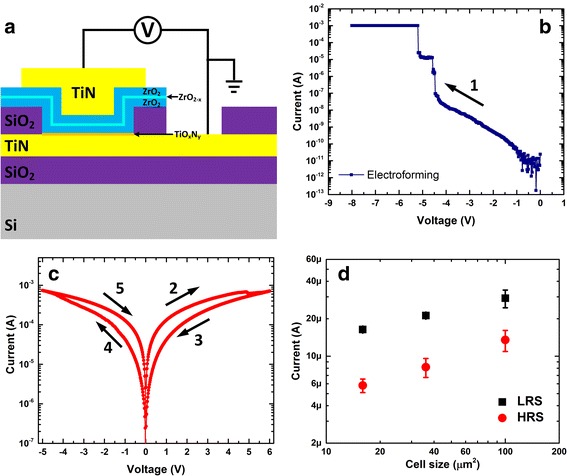
**a** Schematic of the tri-layer TiN/ZrO_2_/ZrO_2 − *x*_/ZrO_2_/TiN memory. **b** *I–V* characteristics of the electroforming process for the TiN/ZrO_2_/ZrO_2 − *x*_/ZrO_2_/TiN device. **c** *I–V* characteristics of the interfacial switching after forming. **d** Current as the function of device size for both HRS/LRS in the interfacial switching mode

Based on the characterization results, the schematic of the ZrO_2_/ZrO_2 − *x*_/ZrO_2_ tri-layer memory is depicted in Fig. 4a. The pristine device is measured to be in the high-resistance state as shown in Fig. 4b. A large negative forming voltage (*I*_CC_ = 1 mA) is required to induce the soft dielectric breakdown and initiate the switching. Quite unusually, this is associated with a two-step forming process, which suggests the consecutive formation of two filaments in the two ZrO_2_ layers and the device is *SET* into a low-resistance state. A positive voltage was then applied to *RESET* the device into the HRS as demonstrated in Fig. 4c. Noticeably, this *RESET* process is characterized by a gradually continuous decrease in current, a typical feature for the interfacial resistive switching. The *SET* process by applying a negative voltage bias also shows the similar behaviour, suggesting the interfacial resistive switching is the dominant switching mode. The interfacial switching behaviour is further proved by the area dependence of current in both HRS and LRS (shown in Fig. 4d). Both currents increase as electrode size increases, indicating the current conduction is not filamentary. The increase in current is though not fully proportional to the area. This could be explained by the less effective modulation of resistivity at bigger cell sizes during the interfacial switching due to the larger amount of grain boundaries and leakage current [12]. Similar behaviour was also reported in other bilayer interfacial resistive switching devices [8, 12]. The *SET* process demonstrates a self-compliance behaviour. This is beneficial for the application in resistive memory as it minimize the current overshoot during switching and has the potential to greatly lower the fabrication cost [29, 30].

**Fig. 5:**
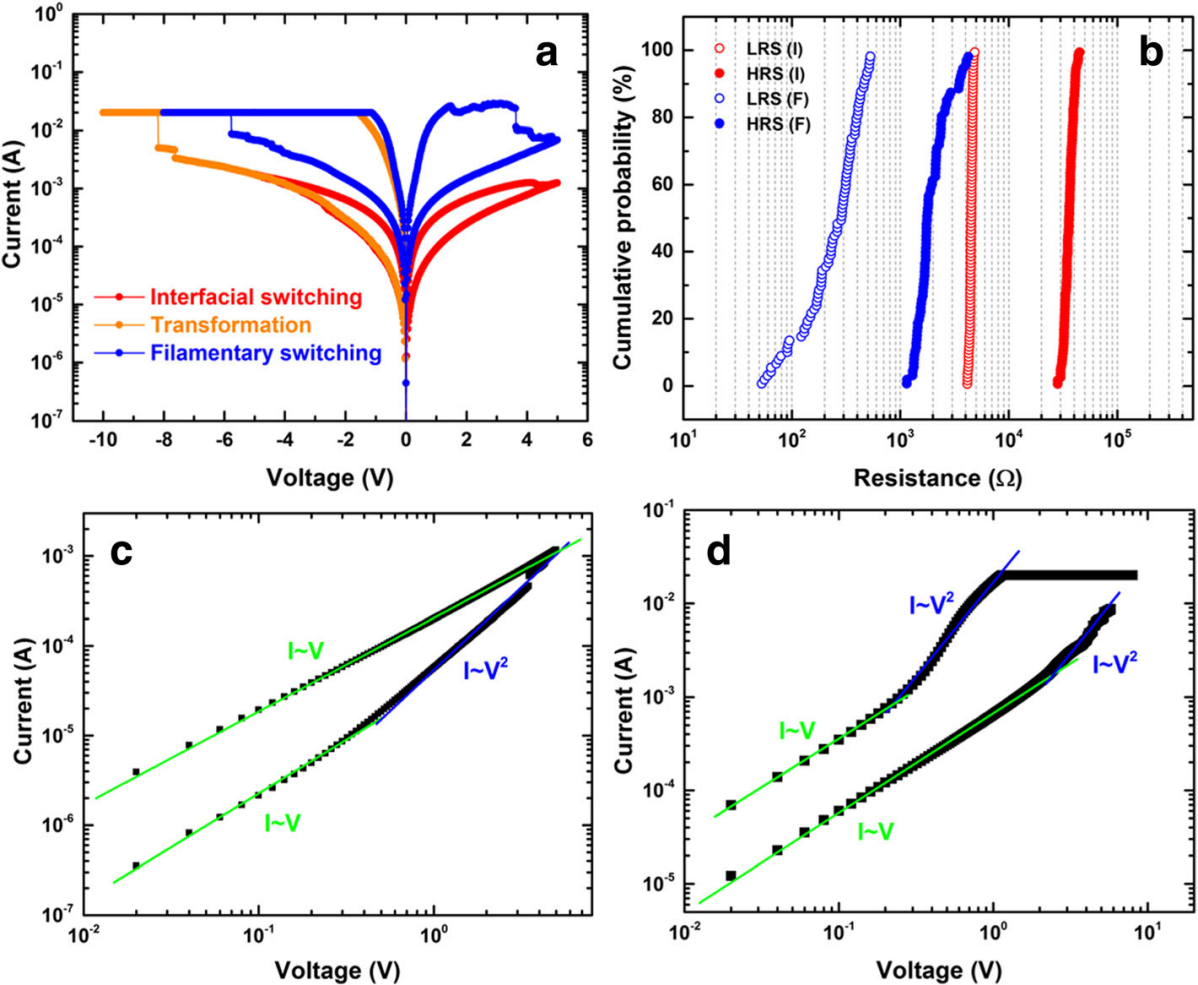
**a** *I*–*V* characteristics of the transformation process (*orange*) from the interfacial switching (*red*) to the filamentary switching (*blue*). **b** Cumulative probability graph of HRS and LRS for both interfacial (*V*_*RESET*_ = 6 V) and filamentary switching modes. The *SET* process *I–V* curves of the **c** interfacial and **d** filamentary switching modes in double-logarithmic plot

The transformation from the interfacial switching mode to the filamentary switching mode can be triggered by a second forming step as demonstrated in Fig. 5a. A more negative bias was applied on the device at HRS with a current compliance of 20 mA. This leads to an abrupt increase of current at ca. −8 V, and the device subsequently remains at a much lower resistance state. After the *RESET* process with a positive bias, the device switching mode has been completely transformed to filamentary, characterized by the sharp *SET* (current control) and *RESET* transitions. Figure 5b shows the cumulative probability distribution of the LRSs and HRSs of both the interfacial and filamentary switching modes in which distinctive differences can be observed between those two sets of resistance states, indicating the device has been switched in different modes. To shed light on the conduction mechanism of both switching modes, the logarithmic *I–V* curve plots and linear fittings of the *SET* processes are presented. The *I–V* curve at HRS in interfacial mode follows an Ohmic behaviour at low voltage with the addition of a quadratic term at higher voltage, i.e., *I* ∝ *aV* + *bV* ^2^, which is the typical feature of the space charge limited current (SCLC) model as shown in Fig. 5c [40–42]. Similar observations of this SCLC mechanism were also reported on other interfacial resistive memory devices [8, 12]. On the other hand, the logarithmic *I–V* curves of the *SET* process in filamentary switching after the transformation is shown in Fig. 5d. The curve suggests the current is governed by the SCLC model with traps [40–42]. Although a similar conduction model is utilized to explain the interfacial and filamentary switching, the two modes still demonstrate distinct features, particularly at a low-resistance state. The current conduction for LRS in filamentary mode consists of two regions: the Ohmic region (*I* ∝ *V*) and the Child’s law region (*I* ∝ *V* ^2^) whereas the latter was not observed in the LRS conduction of interfacial switching. This indicates the filamentary switching is mediated by a carrier trapping/detrapping process [43]. We speculate that a substantial amount of traps are generated in the conductive paths during the second formation process, leading to the quadratic term of current in LRS of the filamentary mode.

**Fig. 6:**
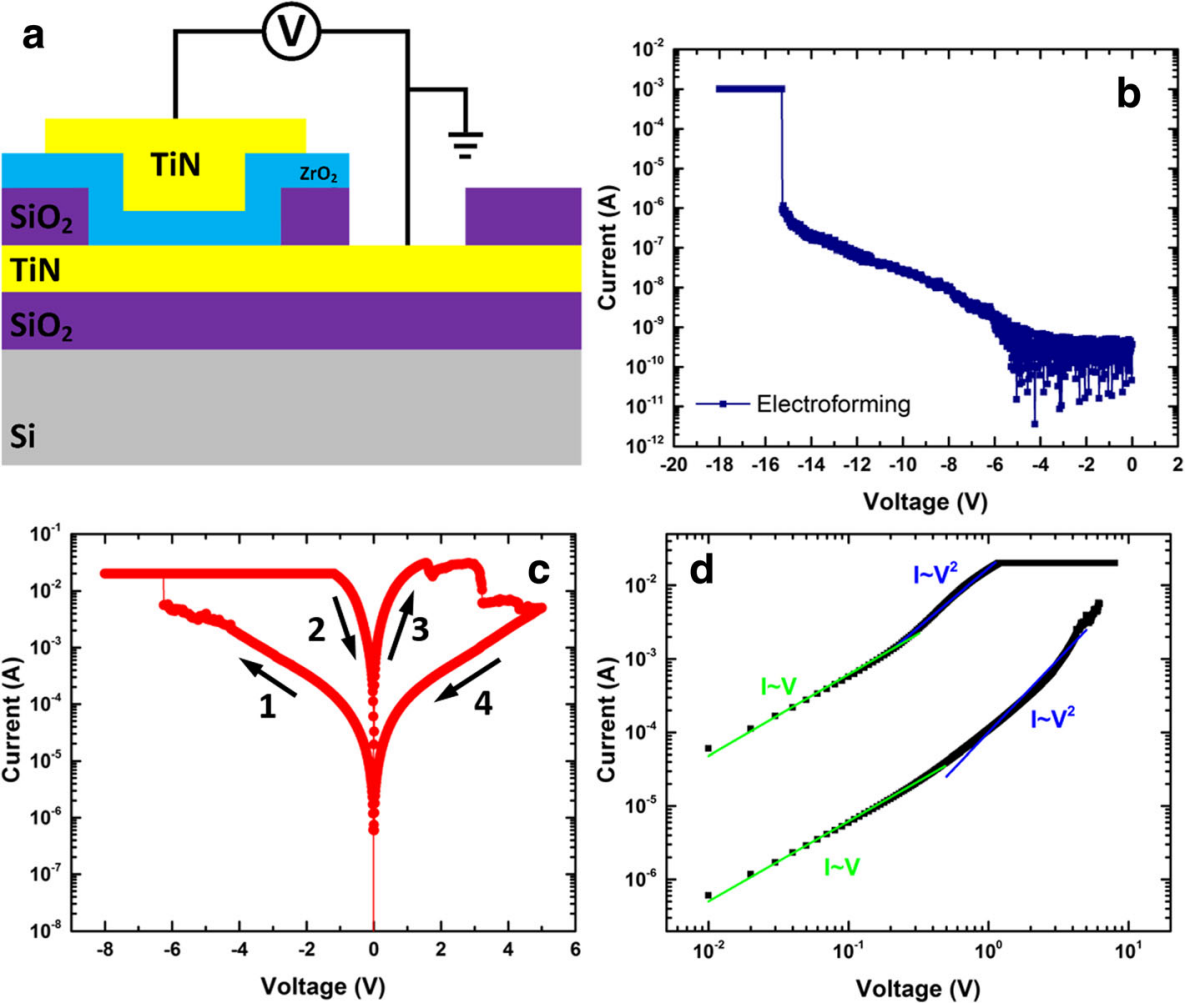
**a** Schematic of the single layer TiN/ZrO_2_/TiN device. **b** *I–V* characteristics of the electroforming process for the TiN/ZrO_2_/TiN device. **c** *I–V* characteristics of the TiN/ZrO_2_/TiN device after forming. **d** *I–V* curves of the *SET* process in double-logarithmic plot with linear fitting

Single-layer ZrO_2_ memory with a film thickness of 40 nm was also fabricated for comparison with the schematic shown in Fig. 6a. The electroforming process (*I*_CC_ = 1 mA) of the TiN/ZrO_2_/TiN device features a single step with much higher voltage (Fig. 6b). Bipolar switching behaviour was subsequently observed (Fig. 6c), which is similar to the filamentary mode in the tri-layer device. However, the interfacial switching mode was not observed in this single-layer device. Figure 6d shows the logarithmic *I–V* curves of the *SET* processes for single-layer devices which demonstrates good agreements with SCLC model with traps. This also support the conclusion that the sandwiched ZrO_2 − *x*_ layer is crucial for the interfacial switching which takes places either at the ZrO_2 − *x*_ layer or near the ZrO_2 − *x*_/ZrO_2_ interface.

**Fig. 7:**
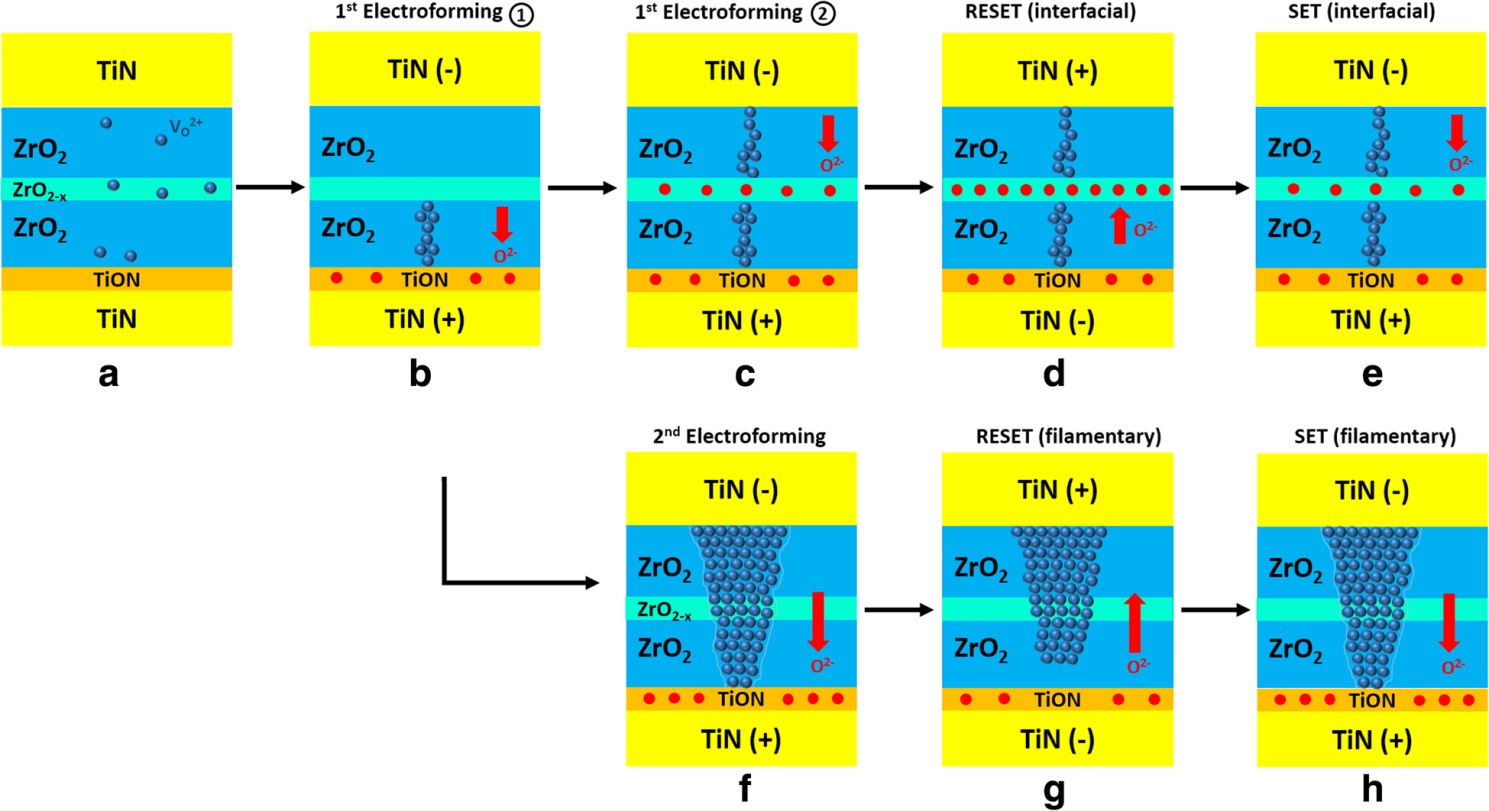
Schematics of the switching mechanism on the tri-layer TiN/ZrO_2_/ZrO_2 − *x*_/ZrO_2_/TiN device for the interfacial switching mode (**a**–**e**). The transformation from interfacial mode to filamentary mode (**f**) and the filamentary mode (**g**, **h**)

Based on the characterization results, a detailed mechanism of both switching modes and the transformation are proposed as shown in Fig. 7. By applying a negative forming voltage, the oxygen ions are pushed down towards the bottom electrode while the oxygen vacancies migrate towards the top electrode and form a conductive filament. The interfacial TiO_*x*_N_*y*_ layer plays a crucial role in the bipolar behaviour as it serves as an oxygen reservoir [38, 44]. Two weak filaments are generated consecutively within the bottom and top ZrO_2_ layers, characterized by the two-step forming process (Fig. 7b, c). Although some oxygen ions might have been injected into the middle ZrO_2 − *x*_ layer, the level of oxygen vacancies is still high enough to keep the layer in a low resistive state. The device has hence been switching into the LRS (Fig. 7c). When a positive bias is applied, oxygen ions are attracted from the TiO_*x*_N_*y*_ layer to the ZrO_2 − *x*_ layer, resulting in the formation of an oxygen-rich layer. This stoichiometric modulation of the ZrO_2 − *x*_ layer changes memory to HRS (Fig. 7d). Another negative bias is required to *SET* the device into LRS by pushing the oxygen ions from the middle layer back to the TiO_*x*_N_*y*_ reservoir (Fig. 7e). The compliance-free property of the device may derive from the low conductivity of the two filaments, which effectively serves as the intrinsic series resistors. The transformation happens when a significantly large bias is applied which induces the formation of a much stronger and conductive filament through the entire tri-layer structure (Fig. 7f). The resistance of the device no longer depends on the ZrO_2 − *x*_ layer, and the switching mode is transformed from interfacial to filamentary. The device can then be switched ON and *OFF* using positive and negative bias, respectively (Fig. 7g, h).

**Fig. 8:**
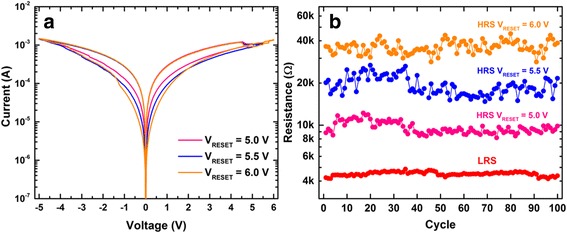
**a** *I–V* characteristics of the interfacial switching for the tri-layer TiN/ZrO_2_/ZrO_2 − *x*_/ZrO_2_/TiN device with varying *RESET* voltages. **b** Endurance test of 100 cycles for the interfacial switching with different *RESET* voltages

The interfacial switching mode in this work offers the potential for multistate storage. As the device resistance in the interfacial mode is mediated by the level of oxygen ions in the ZrO_2 − *x*_ layer, multiply resistance states can be achieved by controlling the amount of oxygen ions in the middle layer using different *RESET* voltages. Figure 8a shows the *I–V* characteristics of the device with *RESET* voltages of 5.0, 5.5 and 6.0 V with a constant *SET* voltage of −5 V. It is worth mentioning that as the migration of oxygen vacancies is driven by the electrical field, these parameters are scalable with layer thickness. With a thick functional layer of around 50 nm, this presented work still has a good potential to scale down and significantly reduce both the *SET* and *RESET* voltages. Figure 8b displays the cycling characteristics of this multistate memory operation where reversible and reproducible resistive switching property is demonstrated for ca. 100 cycles, indicating a promising endurance behaviour of this device.

## Conclusions

A controllable transformation from the interfacial mode to the filamentary mode interfacial mode was observed in a ZrO_2_/ZrO_2 − *x*_/ZrO_2_ tri-layer memory. The possible switching and transformation mechanism is proposed. The embedded ZrO_2 − *x*_ layer is believed to be a crucial layer for the interfacial switching mode. This mode exhibits a compliance-free behaviour possibly due to the intrinsic series resistor by the two thin filaments formed in the ZrO_2_ layers. By controlling the *RESET* voltages, multistate resistances at HRS can be achieved. This multistate storage behaviour clearly points towards the capability of developing the next-generation multistate high-performance memory.

## Additional file

Additional file 1: Supplementary information.

## Abbreviations

ADF-STEM: Annular dark-field scanning transmission electron microscopy
EDX: Energy-dispersive X-ray spectroscopy
HRS: High-resistance state
IRS: Intermediate-resistance state
LRS: Low-resistance state
RRAM: Resistive random access memory
SCLC: Space charge limited current
XPS: X-ray photoelectron spectroscopy
XRD: X-ray diffraction
