# Supplementary material for: Compliance-Free ZrO2/ZrO2 − x/ZrO2 Resistive Memory with Controllable Interfacial Multistate Switching Behaviour
Source: Nanoscale Res Lett. 2017 Jun 2;12:384. doi: 10.1186/s11671-017-2155-0 (PMC5457368; doi:10.1186/s11671-017-2155-0)
Supplement: Additional file 1: — Supplementary information. [file 11671_2017_2155_MOESM1_ESM.pdf]

Supplementary Information

Compositional characterisations were firstly carried out on the as-deposited ZrO_x films with O_2 flow rate ranging from 6 sccm to 20 sccm by EDX as shown in Figure S1. Films with a large thickness of 1 μm were deposited directly onto Si wafers to minimise the influence from the substrate. Sub-stoichiometric ZrO_{2-x} films were obtained when the O_2 flow rate was below 12 sccm and the composition changes from *ca.* $ZrO_{1.2}$ to $ZrO_{1.8}$ with increasing flow rate from 6 sccm to 10 sccm. The O:Zr ratio then saturates when more O_2 is introduced into the chamber and stoichiometric ZrO_2 films were obtained.

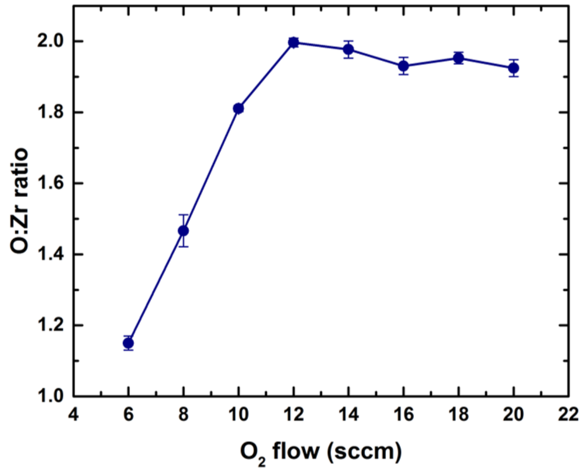

Figure S1. (a) EDX quantitative O:Zr ratio of the as-deposited ZrO_x films with different O_2 flow rates from 6 sccm to 20 sccm.

Figure S2 shows the XRD patterns of the ZrO_x films deposited with different O_2 flow rate. Under the 6 sccm O_2 flow rate, the as-deposited ZrO_x film is found to be amorphous, characterised by the large bump distributed in a wide range. Further oxidization of the ZrO_x film resulted in higher film crystallinity and the films were gradually transformed into the tetragonal phase when the O_2 flow rate reaches 10 sccm. Another phase change to the monoclinic structure was also observed at higher O_2 flow rates (≧ 12 sccm) when stoichiometric ZrO_2 films were obtained.

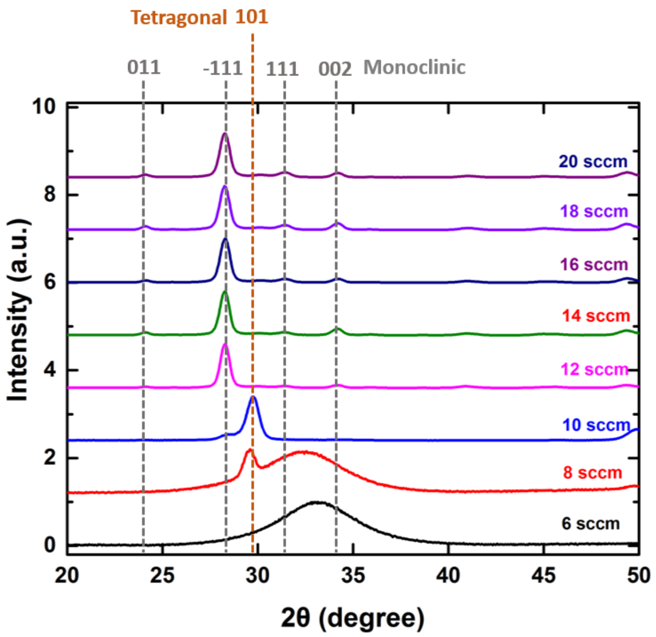

Figure S2. XRD patterns of ZrO_x films deposited at different O_2 flow rates from 6 sccm to 20 sccm.
